# Cheap Oxygen—Ammonia Prepared from the Nitrogen of the Air

**Published:** 1872-07

**Authors:** F. Moigno


### Cheap Oxygen—Ammonia Prepared from the Nitrogen of the Air.

REV. F. MOIGNO.

The excellent author first briefly refers to a paper published in the *Chemical News* of Feb. 23d last, "On the Utilization of Waste Substances in Gas Liquor," by R. F. Smith, and then states that the cyanide of titanium alluded to will become shortly a substance of great industrial importance, inasmuch as this substance will be used in Tessie du Motay's process, after withdrawing oxygen from the air by means of manganate of soda, to fix the nitrogen of the air, and next to convert this into ammonia by passing hydrogen over it; it appears that the experiments made in this direction have been very successful. —*Chemical News, from Les Mondes.*

